# Correction: A Conserved Interaction between a C-Terminal Motif in Norovirus VPg and the HEAT-1 Domain of eIF4G Is Essential for Translation Initiation

**DOI:** 10.1371/journal.ppat.1005509

**Published:** 2016-03-11

**Authors:** Eoin N. Leen, Frédéric Sorgeloos, Samantha Correia, Yasmin Chaudhry, Fabien Cannac, Chiara Pastore, Yingqi Xu, Stephen C. Graham, Stephen J. Matthews, Ian G. Goodfellow, Stephen Curry

The following information is missing from the Funding section: Frédéric Sorgeloos was funded by a BBSRC sLoLa award (reference: BB/K002465/1).
