# Supplementary figures and images for: A Conserved Interaction between a C-Terminal Motif in Norovirus VPg and the HEAT-1 Domain of eIF4G Is Essential for Translation Initiation
Source: PLoS Pathog. 2016 Jan 6;12(1):e1005379. doi: 10.1371/journal.ppat.1005379 (PMC4703368; doi:10.1371/journal.ppat.1005379)

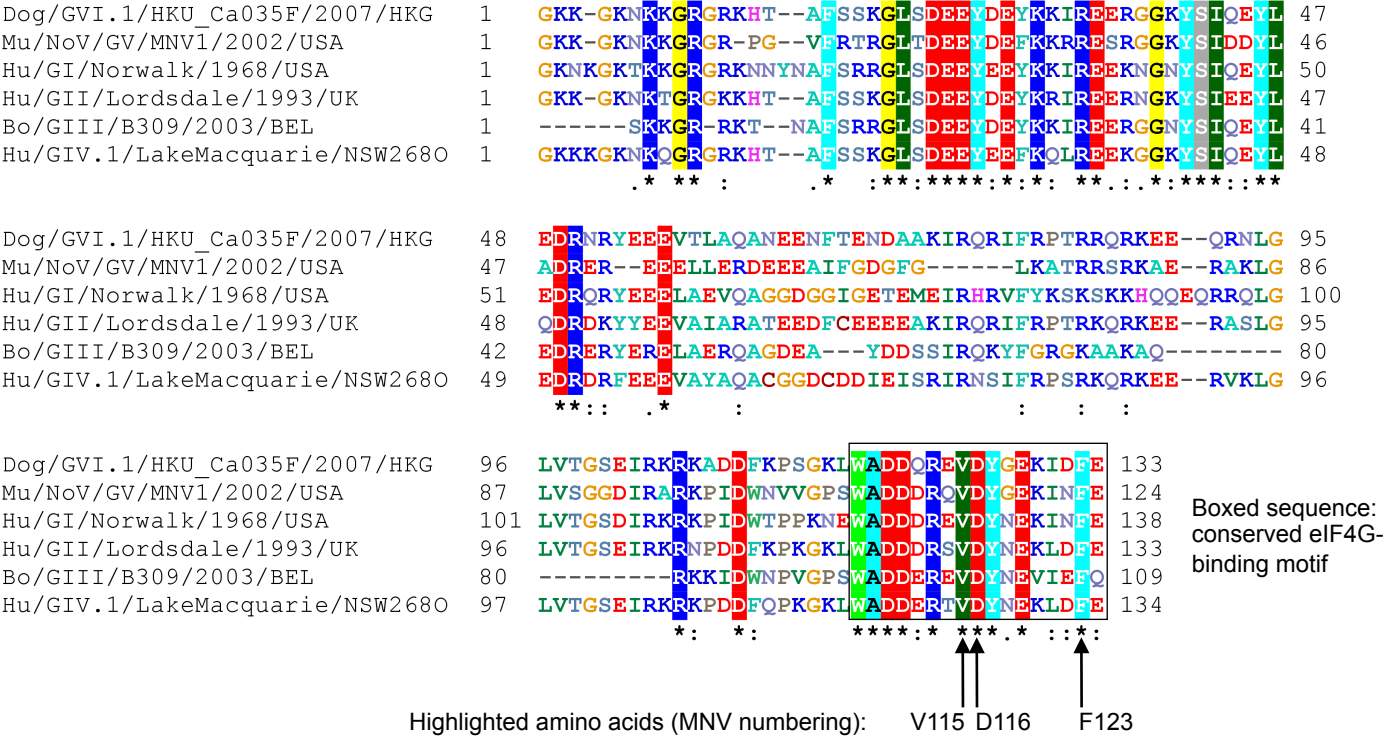

Fig S1

Supplement: S1 Fig — The representative strains used in the alignments are GI Hu/GI/Norwalk/1968/US, (NCBI accession AAC64602), GII Lordsdale virus Hu/GII/Lordsdale/1993/UK (NCBI accession P54634), GIII Bo/GIII/B309/2003/BEL (NCBI accession ACJ04905.1),Hu/GIV.1/LakeMacquarie/NSW268O (NCBI accession number AFJ21375), Mu/NoV/GV/MNV1/2002/USA (NCBI accession ABU55564.1), GVI dog/GVI.1/HKU_Ca035F/2007/HKG (NCBI accession FJ692501). Sequence alignment was performed by ClustalW [67] and BioEdit (http://www.mbio.ncsu.edu/BioEdit/bioedit.html). The conserved eIF4G-binding motif is boxed. Selected amino acids from this motif are indicated (with MNV VPg numbering). (PDF) [file ppat.1005379.s001.pdf]

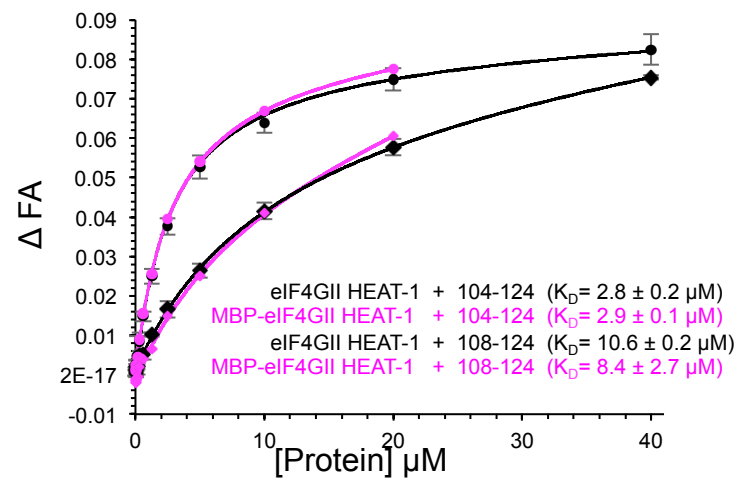

Fig S3

Supplement: S3 Fig — FITC-labelled MNV VPg(104–124) and MNV VPg(108–124) peptides were used in fluorescence anisotropy binding assays with the HEAT-1 domain of eIF4GII (745–1003) in order to determine the KD of the interaction. ΔFP, the normalised change in fluoresce anisotropy (relative to a no protein control) is plotted against protein concentration. Where appropriate (N>1), error bars indicate the standard deviation in the mean ΔFP value observed. The assays were performed with untagged eIF4GII HEAT-1 (black) and an MBP-tagged version (purple). In each case the data were fit using GraphPad Prism to a single-site binding model. (PDF) [file ppat.1005379.s003.pdf]

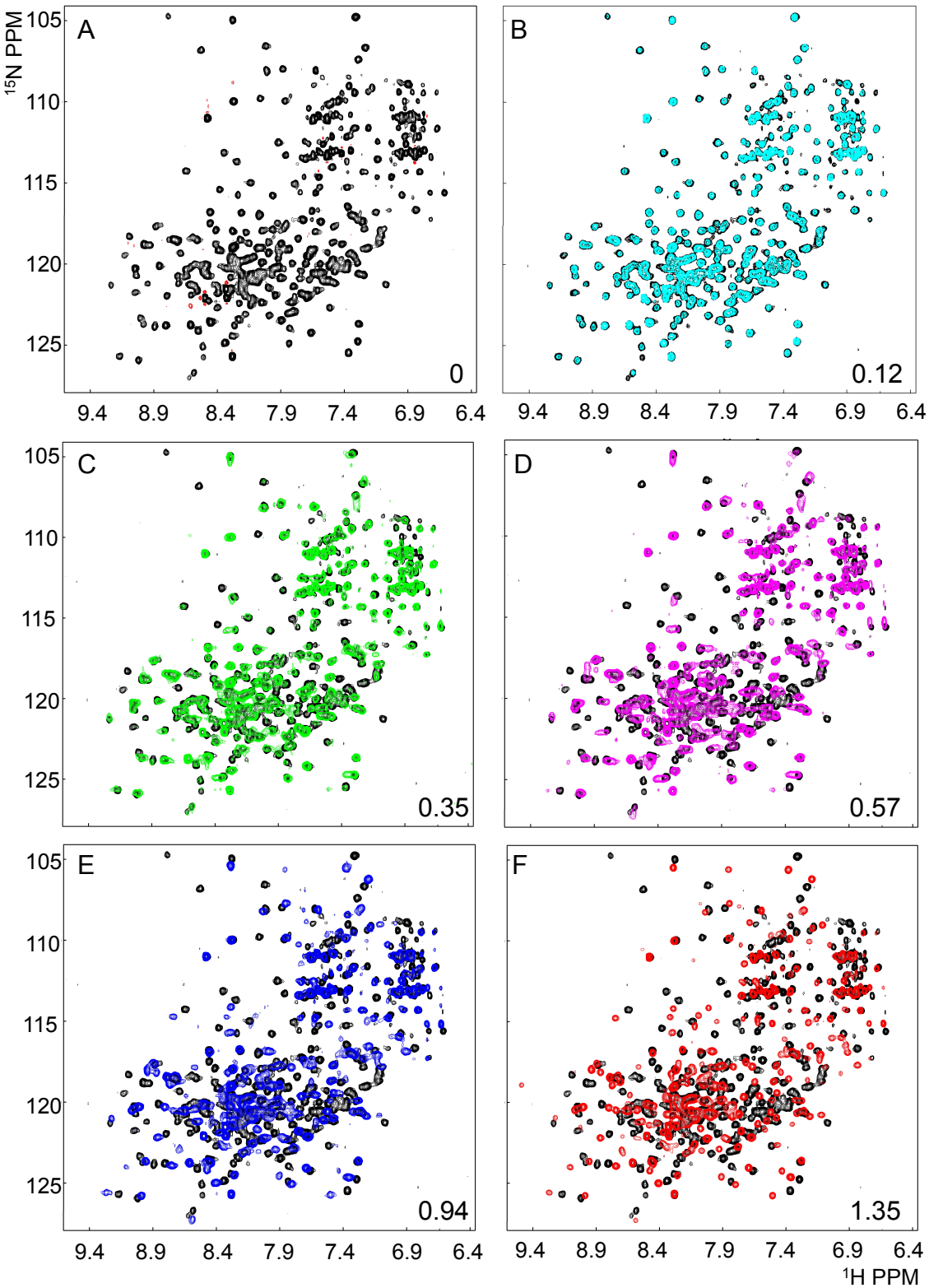

Fig S4

Supplement: S4 Fig — 1H-15N TROSY HSQC spectra obtained on titration of unlabelled MNV VPg(104–124) into 212 μM 15N-labelled eIF4GI HEAT-1 (748–993). (A) Reference spectrum obtained in the absence of MNV VPg(104–124). (B-F) Spectra obtained in the presence of (B) 0.12, (C) 0.35, (D) 0.57, (D) 0.94 and (F) 1.35 molar equivalents of MNV VPg(104–124) peptide superposed on the reference spectrum. The molar equivalents of MNV VPg(104–124) peptide and the number of scans used to obtain the spectrum (which was increased as the average signal intensity decreased) each point in the titration. (PDF) [file ppat.1005379.s004.pdf]

A

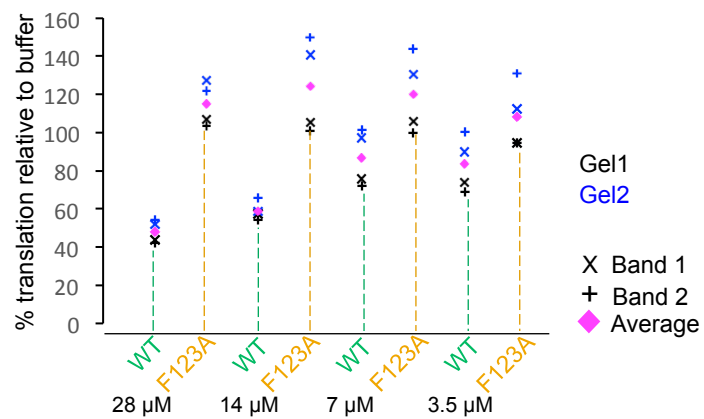

B

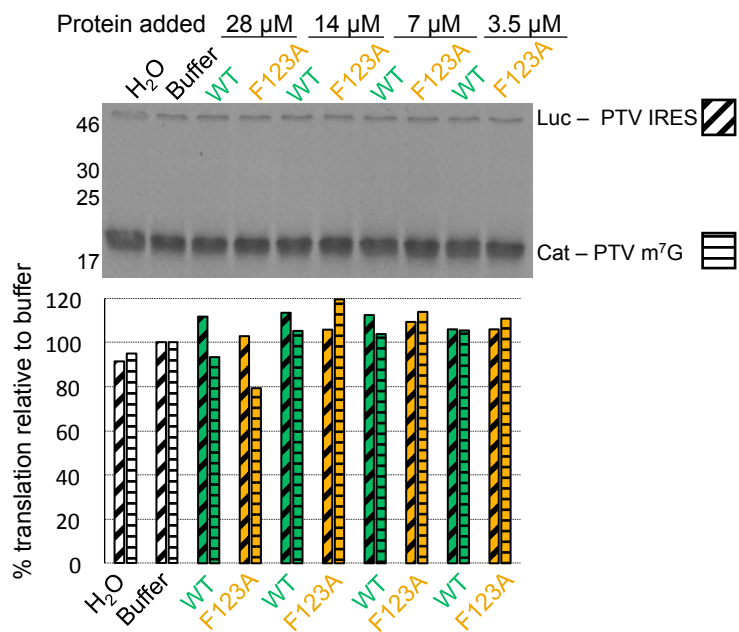

Fig S5

Supplement: S5 Fig — In vitro translation reactions were performed in the presence of increasing concentrations of GST-MNV VPg(102–124) WT protein or the GST-MNV VPg(102–124) F123A mutant that binds much less well eIF4G. Protein synthesis was monitored by autoradiography of SDS PAGE analysis of incorporation of 35S-methionine in translation reactions. Top panel: Effect of exogenous GST-MNV VPg(102–124) proteins on translation from capped bi-cistronic mRNA constructs containing the PTV IRES between the first (CAT) and second (Luc) cistrons; bottom panel: quantitative analysis of the level of 35S-methionine incorporation. (PDF) [file ppat.1005379.s005.pdf]

A

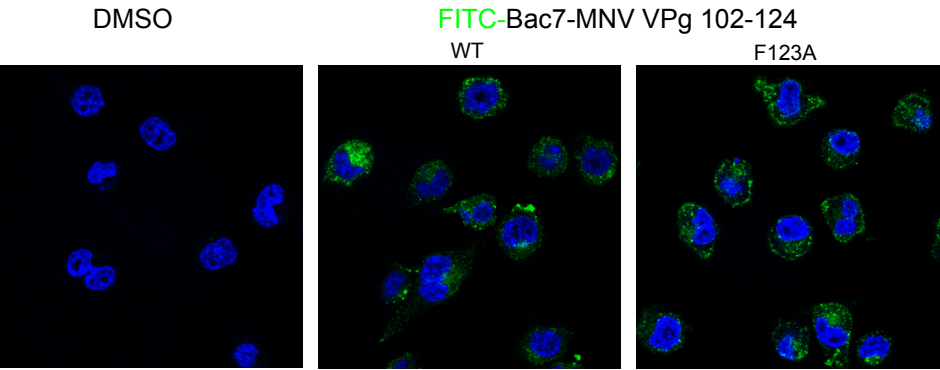

B

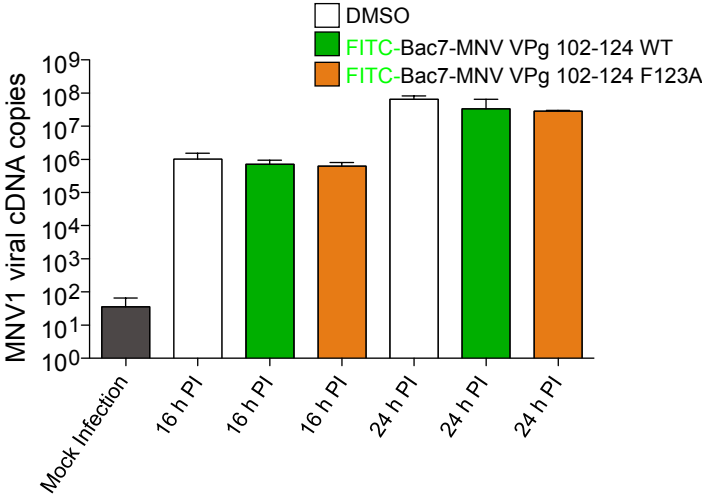

Fig S6

Supplement: S6 Fig — (A) Fluorescence microscopy analysis of cell penetration of the FITC-Bac7-MNV VPg(102–124) peptides. The images shown are merged images of DAPI stained nuclear DNA (blue) and wild-type or F123A versions of the cell penetrating peptides tagged with FITC (green). (B) Time course of BV2 infection (MOI 0.01 TCID50 units/cell) with MNV1 following pre-treatment for 150 minutes with 100 μM of wild-type or F123A versions of the cell penetrating peptides prior to infection. The progress of infection was monitored by RT-PCR analysis (as in Fig 8). (PDF) [file ppat.1005379.s006.pdf]
