# Supplementary material for: A Conserved Interaction between a C-Terminal Motif in Norovirus VPg and the HEAT-1 Domain of eIF4G Is Essential for Translation Initiation
Source: PLoS Pathog. 2016 Jan 6;12(1):e1005379. doi: 10.1371/journal.ppat.1005379 (PMC4703368; doi:10.1371/journal.ppat.1005379)
Supplement: S2 Fig — (A) Schematic of the His-eGFP-VPg|NS6 fusion protein, including the position of TEV NIa cleavage sites and the molecular weights of each of the fused proteins. (B) Auto-cleavage of the eGFP-VPg-NS6 fusion (described in A) in E. coli was monitored by western blotting of SDS PAGE separated E. coli lysate. The membrane was first probed for VPg and subsequently stripped and probed for eGFP. The molecular weight of eGFP-VPg-NS6, eGFP-VPg and VPg are shown on the right in black and are based on the purified recombinant protein run in lanes 6, 7, and 8. Possible cleavage intermediates are shown in red. (PDF) [file ppat.1005379.s002.pdf]

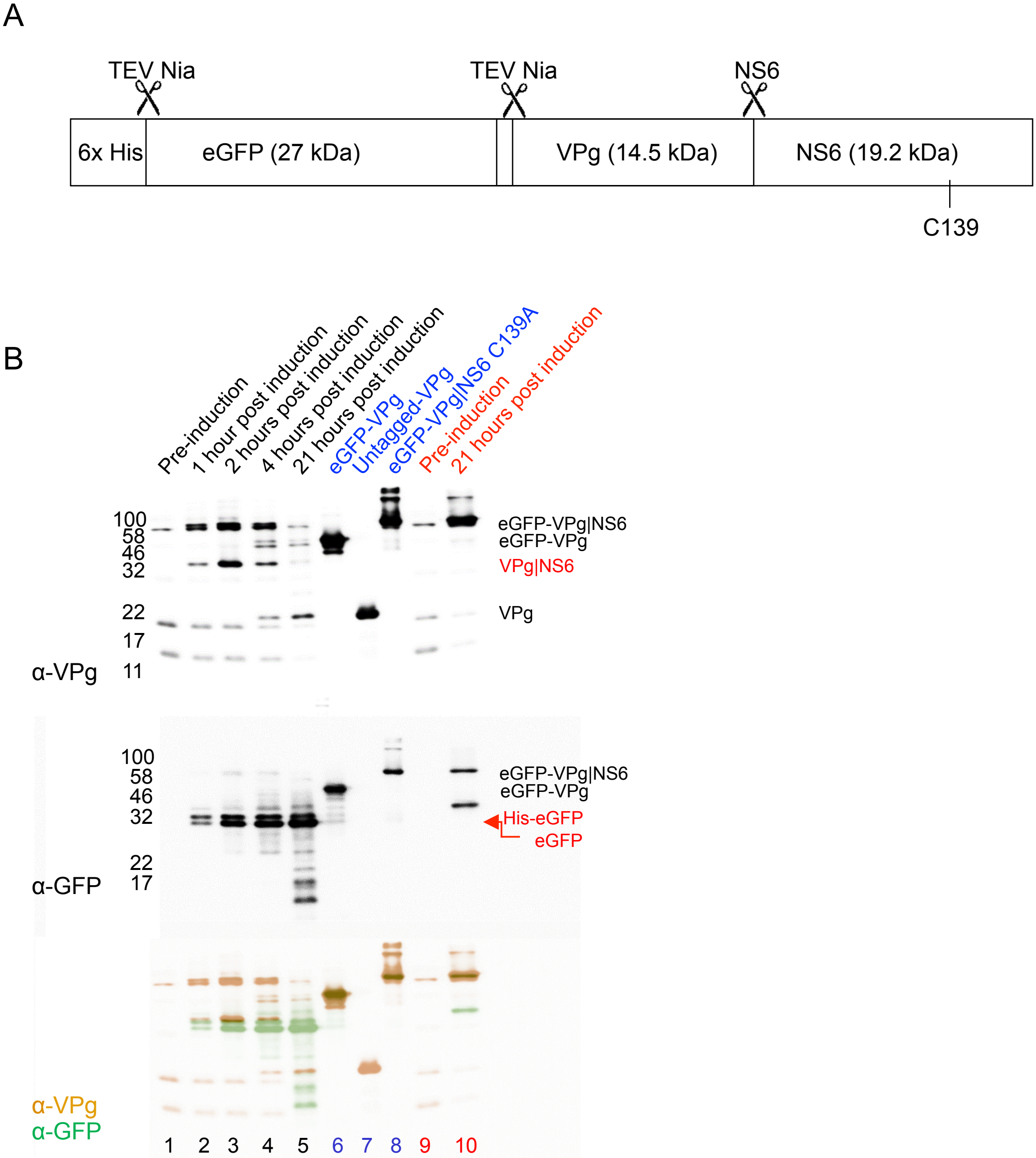

Key to lane labels:

eGFP-NS5|6 WT time course
eGFP-NS5|6 C139A time course
Purified recombinant protein

Fig S2
