# Supplementary material for: A Conserved Interaction between a C-Terminal Motif in Norovirus VPg and the HEAT-1 Domain of eIF4G Is Essential for Translation Initiation
Source: PLoS Pathog. 2016 Jan 6;12(1):e1005379. doi: 10.1371/journal.ppat.1005379 (PMC4703368; doi:10.1371/journal.ppat.1005379)
Supplement: S1 Text — Specific adjustments to purification protocols. (DOCX) [file ppat.1005379.s013.docx]

**A conserved interaction between a C-terminal motif in Norovirus VPg and the HEAT-1 domain of eIF4G is essential for translation initiation**

Eoin N. Leen, Frédéric Sorgeloos, Samantha Correia, Yasmin Chaudhry, Fabien Cannac, Chiara Pastore, Yingqi Xu, Stephen C. Graham, Steve J. Matthews, Ian G. Goodfellow, Stephen Curry

**Supplementary Materials and Methods – Specific adjustments to purification protocols**

**MNV VPg 1-124 WT and mutants used in mutational mapping of the eIF4G-VPg interaction.**

Six of the hexa-His-tagged MNV VPg 1-124 protein constructs (wild-type, F123A, ^120^KIN⭢^120^ESA, ^116^DYGE⭢^116^RAPK, ^112^DRQV⭢^112^REAS, and ^108^WADD⭢^108^APRR) used to delineate the extent of the eIF4GI binding site in cobalt affinity pull-down assays (presented in Fig 2B) were initially purified on TALON resin essentially as described in Materials and Methods. However, following overnight dialysis in 50 mM HEPES pH 7.3, 300 mM NaCl, 2 mM 2-mercaptoethanol, two of the mutant proteins – ^116^DYGE⭢^116^RAPK and ^108^WADD⭢^108^APRR – were found to have OD260/280 ratios of 1.51 and 1.95 respectively. These were unusually high and suggested significant nucleic acid contamination. In order to remove any bound nucleic acids the proteins were diluted approximately ten-fold with phosphate buffer (15.8 mM Na_2_HPO_4_, 34.2 mM NaH_2_PO_4_, pH 6.5, 300 mM NaCl, 1 mM DTT) and protamine sulfate was added to a concentration of 1 mg/mL. Precipitated nucleic acids were removed by centrifugation at 29,000 xg for 20 minutes. The protein in the supernatant was then rebound to TALON cobalt resin (Clontech) for 1 h with slow rotation, after which the slurry was applied to a gravity flow column and the resin washed with 75 mL of phosphate buffer. The protein was eluted with 10 mL of phosphate buffer supplemented with 100 mM imidazole. Eluted fractions were dialysed for 13 hours against 4 L phosphate buffer and the proteins concentrated and frozen as described in the Material and Methods section.

The MNV VPg ^104^VGPS⭢^104^KKAH mutant and ^103^GSGSGS purifications were modified based on the purifications of the other mutants. These were purified as per the Materials and Methods. 50 mM sodium phosphate pH 6.5 (15.8 mM Na_2_HPO_4_, 34.2 mM NaH_2_PO_4_), 300 mM NaCl, 1 mM DTT was used as the buffer for both purification buffer and dialysis.

The final A260/280 of the WT and all the mutant MNV VPg 1-124 proteins was 0.61 or less.

**eIF4GI HEAT-1 truncated (748-993) wild-type and mutants used in mutational mapping of the eIF4G-VPg interaction.**

The wild-type and mutant eIF4GI HEAT-1 (748-993) proteins were initially purified, to completion, as described in Materials and Methods. However the high OD260/280 ratio of some of the mutant proteins was suggestive of nucleic acid contamination (D919R – 1.6, L939A – 1.04, H918A – 1.145, K901M-E914R – 1.15, L897A – 0.84). Therefore the purified proteins were thawed and incubated with 1 µM MgCl_2_ and 387 U benzonase nuclease (Sigma) for 70-90 minutes (typically 1 U benzonase per 0.02 mg protein). The mixture was then subjected to size-exclusion chromatography on a Superdex 75 10/300 GL column equilibrated with 10.1 mM Na_2_HPO_4_, 1.8 mM KH_2_PO_4_, pH 7.2, 136.9 mM NaCl, 2.7 mM KCl, and 1 mM tris(2-carboxyethyl)phosphine hydrochloride (TCEP.HCl). Peak fractions were pooled, concentrated and stored frozen. The final OD260/280 ratios were all under 0.7.
